# Metabolic cell competition in the glioblastoma tumour microenvironment: glucose, glutamine, and lactate as determinants of immune exclusion and targets for pharmacological reprogramming

**DOI:** 10.3389/fonc.2026.1849888

**Published:** 2026-07-14

**Authors:** Egiroh Omene

**Affiliations:** 1 Division of Oncology, Department of Medicine, University of Alberta, Edmonton, AB, Canada; 2 Division of Oncology, Cross Cancer Institute, Edmonton, AB, Canada

**Keywords:** fatty acid oxidation, glioblastoma, glutamine metabolism, glycolysis, metabolic cell competition, pharmacological immunometabolism, tumour microenvironment, tumour-associated macrophages

## Abstract

Glioblastoma (GBM) remains the most lethal primary brain tumour, with median overall survival of 14 to 16 months despite maximal safe surgical resection, concurrent chemoradiotherapy, and adjuvant temozolomide. Treatment failure is driven in large part by a profoundly immunosuppressive tumour microenvironment (TME) in which metabolic competition between GBM cells, bone marrow-derived immunosuppressive myeloid cells, and cytotoxic T lymphocytes determines cellular dominance. This review frames the GBM TME through the lens of metabolic cell competition: a process by which differential metabolic fitness, mediated principally through glucose and glutamine consumption, establishes a suppressive hierarchy that forecloses effective anti-tumour immunity. Aerobic glycolysis in GBM cells produces lactate, which polarises tumour-associated macrophages toward immunosuppressive phenotypes via GPR81/HIF-1*alpha* signalling and directly impairs T cell effector function through extracellular acidification and competition for monocarboxylate transporter capacity. GBM cells and immunosuppressive myeloid cells cannot sustain their proliferative and immunosuppressive programmes without glucose and glutamine; cytotoxic memory T cells, whose effector functions are energetically but not biosynthetically demanding, retain the capacity to function through fatty acid oxidation when these substrates are restricted. Disrupting glucose and glutamine metabolism through glutamine antagonism (DON and prodrugs JHU083/JHU395), dichloroacetate (DCA)-mediated PDK inhibition, intravenous pharmacological ascorbate-mediated GAPDH inactivation and HIF-1alpha destabilisation, systemic glucose restriction (SGLT2 inhibitors), sodium phenylbutyrate-mediated glutamine depletion, and monocarboxylate transporter inhibition can invert this competitive hierarchy, reprogramming the immunosuppressive myeloid compartment while preserving T cell fitness; mebendazole is additionally reviewed as a multi-target anti-parasitic repurposing candidate with demonstrated GBM preclinical survival benefit. Pharmacological ketosis elevates beta-hydroxybutyrate, an endogenous HDAC inhibitor that further augments T cell effector function through NLRP3 inflammasome suppression. The mechanistic and clinical evidence for each intervention is reviewed, metabolic engineering strategies for increasing T cell competitive fitness are described, and principal research gaps are identified. GBM cells and immunosuppressive myeloid cells are proposed to constitute a substrate-dependent competitive coalition whose simultaneous disruption is the central therapeutic proposition reviewed. Evidence is synthesised from *in vitro* metabolic competition experiments, immune-competent murine GBM models, mechanistic pharmacology studies, and early-phase clinical pharmacodynamic data in human GBM.

## 1 Introduction

Glioblastoma is the most common malignant primary brain tumour in adults, with an age-adjusted incidence of approximately 3.2 per 100,000 person-years in the United States and a median age at diagnosis of 64 years (1). Despite representing a minority of all primary brain and central nervous system tumours, GBM accounts for the majority of malignant cases and carries a five-year relative survival rate of less than 5% (1). The current standard of care, established by Stupp et al. in 2005 and since largely unchanged, consists of maximal safe surgical resection followed by concurrent temozolomide and fractionated radiotherapy with six cycles of adjuvant temozolomide, extending median survival from approximately 12 months with radiotherapy alone to 14.6 months (2). Molecular stratification by *MGMT* promoter methylation and *IDH* mutation status, codified in the 2021 WHO classification, has refined prognostic grouping but has not yet translated into substantially improved therapeutic outcomes for *IDH*-wildtype GBM, which constitutes the majority of cases (3). Immunotherapy trials, including checkpoint inhibitors targeting PD-1, and cellular therapies directed at EGFRvIII and IL13R*alpha*2, have yielded uniformly disappointing results in randomised trials, indicating that the immunosuppressive biology of GBM cannot be overcome by targeting a single immune checkpoint or antigen (4).

The failure of immunotherapy in GBM is not primarily attributable to a lack of tumour antigenicity or deficient T cell priming at peripheral lymphoid organs, but rather to the deeply immunosuppressive microenvironment within which primed effector cells must operate. The GBM TME is a densely populated and functionally integrated ecosystem comprising malignant cells of multiple states, tumour-associated macrophages and microglia, myeloid-derived suppressor cells (MDSCs), regulatory T cells (Tregs), exhausted cytotoxic lymphocytes, cancer-associated fibroblasts (CAFs), pericytes, and endothelial cells (5). Tumour-associated macrophages and microglia together constitute 30 to 50% of the total tumour mass in many GBM specimens, establishing the myeloid compartment as the numerically dominant and functionally central cellular element of the TME (6, 7). These cells suppress T cell priming and effector function, promote angiogenesis, remodel the extracellular matrix, and facilitate tumour invasion (8).

One explanation for the intractability of GBM immunosuppression, and for the failure of checkpoint blockade and cellular therapies to overcome it, is that GBM cells and tumour-associated myeloid cells collectively outcompete infiltrating T lymphocytes for limiting metabolic resources within the shared tumour niche, establishing a competitive hierarchy in which immune effector cells are systematically deprived of the biosynthetic and energetic substrates required for their anti-tumour programmes. Cell competition, first described in *Drosophila melanogaster* by Morata and Ripoll and subsequently characterised across mammalian developmental contexts, refers to the selective elimination of cells with reduced fitness by neighbouring cells with superior fitness within a shared niche. In the canonical developmental setting, cells with lower Myc expression or altered Flower isoform signalling are recognised as “losers” and eliminated through contact-dependent mechanisms, secreted factors, or metabolic signals, maintaining tissue quality. In cancer, this concept has been extended to describe how tumour cells outcompete normal stromal and epithelial neighbours during oncogenesis, and more recently how tumour cells and immunosuppressive myeloid cells collectively outcompete anti-tumour effector lymphocytes for survival signals and metabolic substrates within the TME (5, 9).

The molecular bridge between canonical cell competition and the GBM metabolic context is c-Myc. In canonical cell competition, cells with higher Myc activity consistently outcompete and eliminate lower-Myc neighbours through contact-dependent and paracrine mechanisms, a phenomenon characterised as supercompetition and demonstrated in mammalian developmental contexts by Clavería et al. (10) In GBM, *MYC* transcriptional activation downstream of EGFR/PI3K/mTOR signalling is frequent in *IDH*-wildtype disease; focal *MYC* gene amplification is present in approximately 5 to 10% of cases and should be distinguished from the more prevalent transcriptional upregulation. Myc directly drives transcription of the glycolytic and glutamine metabolic programmes, including *GLUT1*, *HK2*, *LDHA*, and *GLS1*, and this transcriptional output is the mechanism by which Myc-high cells achieve competitive superiority over their neighbours (11). Metabolism is not an analogy for competitive fitness in the GBM TME: it is the molecular mechanism by which fitness is enacted. The glucose consumption rate of a GBM cell, its glutamine catabolism, and the lactate it exports as the obligate byproduct of aerobic glycolysis determine whether neighbouring cells, including infiltrating T lymphocytes, can survive, proliferate, and execute effector programmes within the shared niche. The glucose-glutamine-lactate axis is the molecular substrate of competitive dominance in the GBM immune context, and Myc-driven transcriptional activation of glycolytic and glutaminolytic programmes is the mechanism through which competitive fitness differences between GBM cells and T lymphocytes are produced.

In this review, metabolic cell competition in the GBM TME is defined operationally as the differential consumption of limiting substrates, principally glucose and glutamine, by cells with constitutively active versus regulated metabolic programmes, such that GBM cells and M2-polarised immunosuppressive myeloid cells establish dominance over infiltrating T lymphocytes through substrate depletion, lactate-mediated paracrine immunosuppressive signalling, and amino acid catabolism. This definition preserves the winner/loser structure of canonical cell competition while specifying the operative mechanism: competitive fitness is determined by the rate of substrate acquisition from the shared niche and the capacity to convert this acquisition into proliferative advantage and paracrine suppression of neighbouring competitors. Substrate depletion and inhibitory receptor signalling (PD-1/PD-L1, TIM-3, LAG-3) are not mutually exclusive mechanisms: metabolic starvation contributes to and potentiates the exhaustion phenotype driven by inhibitory receptor engagement, as demonstrated by Bengsch et al., who showed that PD-1 signalling impairs both glycolytic and mitochondrial metabolic capacity, establishing exhaustion as a metabolic state sustained by co-occurring substrate scarcity (12).

Among the mechanisms by which GBM establishes competitive dominance over anti-tumour immune cells, metabolic competition for glucose and glutamine has emerged as both mechanistically central and therapeutically tractable. The Warburg effect, aerobic glycolysis producing lactate regardless of oxygen tension, confers on GBM cells an enormous capacity for glucose consumption that depletes the shared TME of this critical substrate (13, 14). Glutamine, required for nucleotide biosynthesis, TCA cycle anaplerosis, glutathione synthesis, and hexosamine production, is similarly consumed at high rates by both GBM cells and immunosuppressive myeloid cells, creating competitive substrate scarcity that impairs cytotoxic T lymphocyte (CTL) biosynthetic and energetic capacity (15, 16). The obligate byproduct of aerobic glycolysis, lactate, is not merely a metabolic waste product but a paracrine immunosuppressive signal that reprogrammes myeloid cells toward anti-inflammatory phenotypes via GPR81/HIF-1*alpha* signalling and directly suppresses T cell effector cytokine production through extracellular acidification and monocarboxylate transporter competition (17).

**Figure 1:**
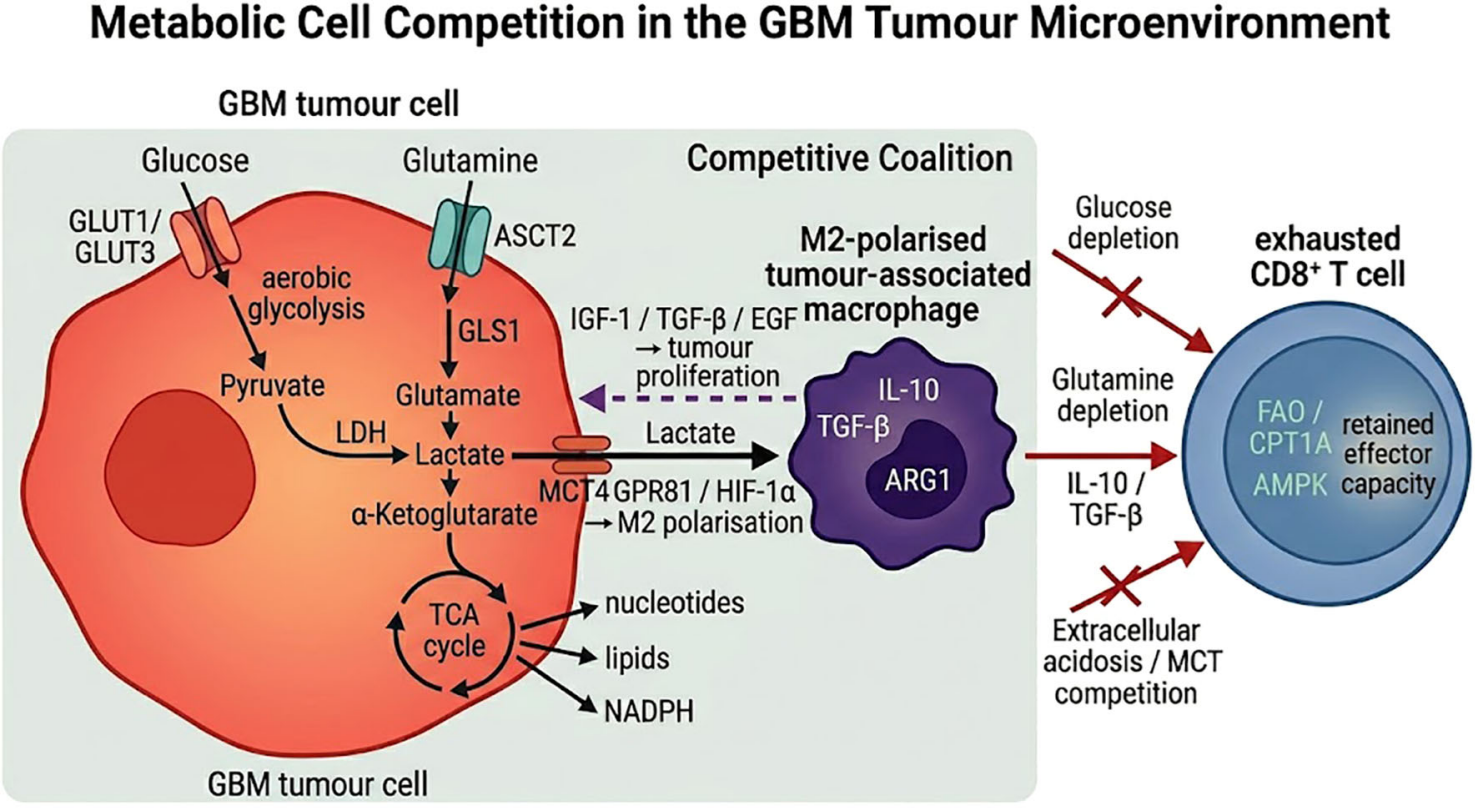
Metabolic cell competition in the glioblastoma tumour microenvironment. The GBM tumour cell and M2-polarised tumour-associated macrophage (TAM) form a competitive coalition (shaded region) through reciprocal metabolic and paracrine interactions that collectively exclude CD8+ T lymphocytes from productive anti-tumour function. Glucose is imported into the GBM cell via GLUT1 and GLUT3 transporters and undergoes aerobic glycolysis (glucose → pyruvate → lactate via LDH). Lactate is exported via MCT4 and binds GPR81 on TAMs, stabilising HIF-1α to drive M2 polarisation; the resultant M2 TAMs secrete IGF-1, TGF-β, and EGF, which feed back to promote GBM tumour cell proliferation (dashed arrow). Glutamine is imported via ASCT2 and catabolised by glutaminase 1 (GLS1) to glutamate, then to alpha-ketoglutarate (α-KG), the principal anaplerotic input to the TCA cycle, supporting nucleotide, lipid, and NADPH biosynthesis. M2-polarised TAMs express IL-10, TGF-β, and arginase-1 (ARG1), mediating direct suppression of T cell effector function. The exhausted CD8+ T cell (right) is deprived of glucose and glutamine by competitive consumption within the TME, is exposed to extracellular acidosis from lactate accumulation and monocarboxylate transporter competition, and receives direct suppressive signals from TAM-derived IL-10 and TGF-β. Despite this suppressive environment, CD8+ T cells retain residual effector capacity through fatty acid oxidation (FAO) mediated by carnitine palmitoyltransferase 1A (CPT1A) and AMPK-driven metabolic reprogramming, defining the therapeutic asymmetry targeted by metabolic competition interventions. α-KG, alpha-ketoglutarate; AMPK, AMP-activated protein kinase; ARG1, arginase-1; ASCT2, alanine-serine-cysteine transporter 2; CPT1A, carnitine palmitoyltransferase 1A; EGF, epidermal growth factor; FAO, fatty acid oxidation; GLS1, glutaminase 1; GLUT1/GLUT3, glucose transporters 1 and 3; GPR81, G protein-coupled receptor 81; HIF-1α, hypoxia-inducible factor 1-alpha; IGF-1, insulin-like growth factor 1; IL-10, interleukin-10; LDH, lactate dehydrogenase; MCT4, monocarboxylate transporter 4; TAM, tumour-associated macrophage; TCA, tricarboxylic acid; TGF-β, transforming growth factor beta; TME, tumour microenvironment.

In the GBM TME, the differential capacity of tumour cells, immunosuppressive myeloid cells, and cytotoxic T cells to sustain their respective programmes under glucose and glutamine restriction determines competitive outcome. GBM cells and immunosuppressive macrophages require glucose and glutamine to sustain proliferation and immunosuppressive function; cytotoxic memory T cells, whose effector programmes are energetically but not biosynthetically demanding, can sustain function through fatty acid oxidation when glucose and glutamine are restricted. Targeted disruption of glucose and glutamine availability therefore selectively disadvantages the tumour-myeloid competitive coalition while preserving T cell fitness. The central mechanistic model is summarised in **Figure 1**. This review is scoped to IDH-wildtype GBM; IDH-mutant gliomas engage oncometabolite-driven immune suppression mechanisms distinct from those reviewed here and require separate evaluation (18, 19). In this review, we examine the evidence that metabolic cell competition drives GBM immunosuppression, evaluate the pharmacological strategies available to invert this competitive hierarchy, and identify the key mechanistic and clinical translation gaps that must be addressed before metabolic immunotherapy can be evaluated in prospective GBM trials.

## 2 The GBM tumour microenvironment: cell types and competitive identities

### 2.1 GBM tumour cells: molecular subtypes and metabolic rewiring

GBM is classified under the 2021 WHO criteria as a diffuse glioma, *IDH*-wildtype, with methylation of the *TERT* promoter, homozygous deletion of *CDKN2A/B*, or chromosome 7 gain and chromosome 10 loss serving as defining molecular features (3). Single-cell RNA sequencing analyses, most comprehensively demonstrated by Neftel et al., have resolved the intratumoral heterogeneity of GBM into four principal cellular meta-states: astrocyte-like (AC-like), mesenchymal-like (MES-like), neural progenitor cell-like (NPC-like), and oligodendrocyte progenitor cell-like (OPC-like), which coexist within individual tumours and interconvert in response to microenvironmental cues (9). The mesenchymal state, enriched in hypoxic and necrotic regions, is associated with the most pronounced immunosuppressive programme and the highest expression of glycolytic enzymes, while the NPC-like state shows relatively greater reliance on oxidative phosphorylation. This metabolic heterogeneity within the tumour cell compartment itself has implications for the uniformity of metabolic targeting strategies and must be accounted for in therapeutic design.

### 2.2 Glioma stem cells: metabolic distinctiveness and therapeutic implications

Glioma stem cells (GSCs), identified by expression of CD133, SOX2, NESTIN, and OLIG2, represent a self-renewing subpopulation that is disproportionately responsible for treatment resistance and post-treatment recurrence (9). Quiescent GSCs maintain lower glycolytic rates and higher mitochondrial activity than differentiated bulk tumour cells, with greater resistance to acute glucose deprivation (20). However, the transition from quiescent to aggressively proliferating GSC is accompanied by upregulation of glycolysis and glutaminolysis to meet the biosynthetic demands of rapid division, including nucleotide synthesis via the pentose phosphate pathway and glutamine-dependent nitrogen donation. The proliferative GSC phenotype responsible for invasion and recurrence is therefore metabolically dependent on glucose and glutamine in the same way as bulk tumour cells.

### 2.3 Tumour-associated macrophages and microglia: the dominant suppressive compartment

The myeloid compartment of the GBM TME comprises two ontologically distinct populations: resident brain macrophages (microglia), derived from yolk sac progenitors that populate the CNS during embryonic development, and bone marrow-derived macrophages (BMDMs) that infiltrate from the periphery in response to tumour-derived chemotactic signals including CCL2, CSF1, and VEGF (6, 7). Resident microglia are characterised by expression of TMEM119, P2RY12, CX3CR1, and SALL1, markers that are progressively lost as microglia adopt tumour-associated phenotypes (7). BMDMs, which enter the CNS parenchyma by traversing a tumour-compromised blood-brain barrier, are identifiable by expression of CD49d and CD38 and by the absence of homeostatic microglial markers (7). Bowman et al. demonstrated that the BMDM fraction increases substantially following radiotherapy and temozolomide treatment, indicating that standard-of-care therapy itself remodels the myeloid composition of the TME in ways that may paradoxically augment immunosuppression (7).

Both resident microglia and BMDMs adopt immunosuppressive phenotypes within the GBM TME, characterised by high expression of IL-10, TGF-*beta*, arginase-1, CD206, and PD-L1, and by reduced capacity for antigen presentation, T cell priming, and pro-inflammatory cytokine secretion (8). However, collapsing these populations into a single “macrophage” category obscures functionally important distinctions: microglial responses are shaped by their developmental origin and CNS-specific sensing machinery, while BMDMs are more dynamically responsive to systemic metabolic and cytokine cues. The M1/M2 dichotomy, while conceptually useful, is an oversimplification of a continuous polarisation spectrum and is increasingly replaced by single-cell resolution descriptions of macrophage states (5). Both populations, in their tumour-associated immunosuppressive states, rely on glycolysis and oxidative glutamine metabolism for their suppressive programmes (21).

### 2.4 T cells, NK cells, and the suppressed effector compartment

CD8+ cytotoxic T lymphocytes and CD4+ helper T cells infiltrate GBM but are present at substantially lower densities than myeloid cells and are functionally impaired by multiple overlapping mechanisms (5). Tumour-infiltrating CD8+ T cells in GBM typically display a phenotype of exhaustion, characterised by co-expression of PD-1, TIM-3, LAG-3, and TIGIT, reduced IFN-*gamma* and perforin secretion, and impaired proliferative capacity (4). NK cells, which do not require MHC-I-restricted antigen presentation for tumour recognition and killing, are similarly impaired by the acidic, lactate-rich, immunosuppressive cytokine milieu of the GBM TME. The metabolic starvation imposed by glucose and glutamine depletion in the TME is mechanistically integrated with this phenotypic exhaustion: T cells deprived of glucose and glutamine cannot sustain the biosynthetic and energetic demands of effector cytokine production, clonal expansion, and cytotoxic granule secretion, as documented in direct competition experiments by Chang et al. and Ho et al. (22, 23).

### 2.5 MDSCs, regulatory T cells, neutrophils, and dendritic cells

MDSCs, comprising both monocytic (M-MDSC) and polymorphonuclear (PMN-MDSC) subsets, accumulate in GBM through CSF1 and IL-6 signalling and suppress T cells through arginase-1-mediated arginine depletion, reactive oxygen species production, and expression of immunosuppressive cytokines (24). Regulatory T cells are expanded in GBM partly through tumour-derived TGF-*beta* and through lactate-dependent stabilisation of *FOXP3* expression (25). Plasmacytoid and conventional dendritic cells are present at low density in GBM and display impaired maturation and antigen cross-presentation capacity, limiting their ability to prime naive T cells in tumour-draining lymph nodes. Tumour-associated neutrophils in GBM, like their myeloid counterparts, adopt a predominantly tumour-promoting phenotype that supports angiogenesis and suppresses lymphocyte activity (5).

### 2.6 Cancer-associated fibroblasts, pericytes, and the stromal metabolic contribution

While GBM lacks the prominent desmoplastic stroma of many extracranial solid tumours, perivascular CAF-like cells and pericytes contribute to the metabolic TME by secreting lactate and amino acid-depleting enzymes, establishing hypoxic gradients through abnormal vasculature, and producing extracellular matrix components that limit immune cell infiltration (5). The tumour vasculature in GBM, which is markedly abnormal and characterised by endothelial proliferation, is a major source of VEGF-driven immunosuppression and represents a site at which lactate export via the vasculature influences the systemic metabolic milieu. Single-cell and spatial transcriptomic studies have begun to map the precise anatomical and functional organisation of these non-immune, non-tumour cell populations within the GBM ecosystem, revealing that they occupy discrete metabolic niches at the tumour-brain interface and around the necrotic core (26, 27).

## 3 Metabolic mechanisms of tumour competitive dominance

### 3.1 The Warburg effect in GBM: glucose consumption and competitive exclusion

Aerobic glycolysis, the preferential conversion of glucose to lactate regardless of oxygen availability, was described in tumour tissue by Warburg in 1927 and subsequently elaborated as a fundamental property of proliferating cells by Vander Heiden, Cantley, and Thompson (13, 14). In GBM, the Warburg effect is driven by oncogenic activation of PI3K/Akt/mTOR signalling, upregulation of GLUT1 and GLUT3 glucose transporters, HIF-1*alpha*-mediated transcription of glycolytic enzymes, and, in a subset of cases, amplification of *EGFR* with downstream metabolic consequences. The consequence of this glycolytic programme is that GBM cells consume glucose at rates vastly exceeding the oxidative capacity of the tissue, generating a glucose-depleted, lactate-rich extracellular environment. F-FDG-PET imaging of GBM demonstrates high metabolic activity in viable tumour regions that typically exceeds that of adjacent normal brain, providing clinical evidence for this avid glucose consumption. Direct in-patient confirmation of active substrate flux was provided by Maher et al., who infused patients with uniformly ^13^C-labelled glucose intraoperatively and demonstrated by NMR spectroscopy that GBM tissue actively converted labelled glucose to lactate, glutamate, and TCA intermediates *in vivo*, establishing that the metabolic substrate competition inferred from cell-line and murine studies operates in the human tumour (28). Within the tumour milieu, this creates the first level of metabolic cell competition: tumour cells that have constitutively activated glycolytic programmes outcompete cells, including T cells, that depend on regulated glucose uptake for their metabolic needs. *In vivo* evidence for this nutrient partitioning hierarchy has been provided by Reinfeld et al., who used radiolabelled PET tracer substrates combined with flow-sorted cell-population analysis across multiple solid tumour models to demonstrate that myeloid cells are the dominant intratumoral consumers of glucose — with T cells intermediate and cancer cells consuming the least glucose — while cancer cells are the dominant consumers of glutamine; this cell-type-specific substrate partitioning directly establishes that differential metabolic acquisition by distinct intratumoral populations operates at the *in vivo* level and supports the competitive coalition model in which the myeloid and cancer cell compartments together deplete the substrates upon which T cells depend (29).

The competitive significance of GBM glucose consumption is amplified by an equally important and less often emphasised dependency: the immunosuppressive myeloid compartment requires glucose to sustain its own suppressive programme. Tumour-associated macrophages in their M2-like immunosuppressive state rely on hexosamine biosynthesis, fuelled by glucose-derived UDP-GlcNAc, to N-glycosylate immunosuppressive surface proteins including CD206, PD-L1, and CD163, maintaining the molecular display that prevents T cell activation and blocks antigen presentation. MDSCs require glucose to sustain the NADPH oxidase-mediated reactive oxygen species production responsible for T cell suppression, and to maintain the arginase-1 expression that depletes arginine from the TME. Both myeloid populations therefore consume glucose in service of their immunosuppressive programmes, compounding the tumour cell glucose sink. The result is a competitive coalition in which two numerically dominant cell types, GBM cells and immunosuppressive myeloid cells, consume the shared glucose supply from the same niche, leaving T cells metabolically marginalised. When glucose is abundant, this coalition is stable and self-reinforcing: the myeloid compartment can sustain its suppressive transcriptional programme while GBM cells proliferate. When glucose is depleted, both pillars weaken simultaneously: GBM proliferation slows, the myeloid immunosuppressive programme loses its metabolic substrate, and the competitive pressure on T cells eases at multiple nodes concurrently.

### 3.2 Glutamine metabolism: biosynthetic dominance and competitive depletion

Glutamine is the most abundant circulating amino acid and the predominant anaplerotic substrate for the TCA cycle in rapidly proliferating cells. In GBM cells, glutamine catabolism via glutaminase (GLS1/GLS2) to glutamate and subsequently to alpha-ketoglutarate supports TCA cycle flux, enabling continued mitochondrial ATP production and biosynthetic intermediate generation under conditions of glucose restriction (15). Beyond energy metabolism, glutamine is the nitrogen donor for *de novo* nucleotide biosynthesis (purine and pyrimidine rings), the precursor for glutathione synthesis that enables redox homeostasis, and the substrate for hexosamine biosynthesis supporting glycoprotein production (15, 16). Tardito et al. demonstrated that GBM cells can upregulate glutamine synthetase to produce endogenous glutamine, enabling partial independence from exogenous supply and representing an adaptive resistance mechanism to glutamine restriction (16). The immunological consequence of high glutamine consumption by both GBM cells and tumour-associated myeloid cells is competitive depletion of this substrate from the TME, depriving T cells of the glutamine required for their own nucleotide biosynthesis, cytokine production, and effector programme execution.

The myeloid compartment’s glutamine dependency is as fundamental as its glucose dependency and deserves explicit attention. M2-polarised TAMs require glutamine for polyamine biosynthesis via the arginase-ornithine-ornithine decarboxylase pathway; these polyamines, including putrescine, spermidine, and spermine, regulate chromatin accessibility at M2 loci and are essential for sustaining the M2 transcriptional programme. Glutamine-derived alpha-ketoglutarate provides the principal anaplerotic input to the TCA cycle in M2 macrophages that preferentially use OXPHOS, and disrupting this input collapses mitochondrial metabolism in the immunosuppressive myeloid cell in the same way that glucose restriction collapses it in glycolytic GBM cells. MDSCs require glutamine for their expansion in response to tumour-derived CSF1 and IL-6, and for the sustained suppressive activity that depends on arginine depletion and ROS generation. Leone et al.’s demonstration that DON-mediated pan-glutamine antagonism reprogrammed TAMs from immunosuppressive to pro-inflammatory phenotypes and restored CD8+ T cell infiltration and IFN-*gamma* production provides direct experimental evidence that the myeloid immunosuppressive programme is glutamine-dependent (30). Glutamine restriction simultaneously deprives GBM cells of nucleotide and glutathione biosynthetic substrate, collapses the myeloid immunosuppressive transcriptional programme, and preferentially spares the anti-tumour immune compartment, whose memory T cell component can compensate through FAO-based energy production without losing effector function. Mechanistic evidence for glutamine flux in the intact GBM TME has recently been extended by Savani et al., who performed ^15^N_2_-glutamine stable isotope tracing in IDH-wildtype GBM tissue explants cultured in human plasma-like medium, demonstrating TME-dependent glutamine metabolic features not captured in monoculture models, including immunomodulatory purine synthesis from glutamine-derived nitrogen and transcriptional correlates of glutamine catabolism identified by spatial transcriptomics (31).

### 3.3 Lactate as the central paracrine immunosuppressive signal

Lactate, exported from glycolytic GBM cells primarily through the monocarboxylate transporter MCT4 (SLC16A3), accumulates in the GBM TME at concentrations of 10 to 30 mM in tumour tissue compared with 1 to 2 mM in normal brain parenchyma (32). High lactate concentrations in solid tumours predict adverse clinical outcomes across multiple cancer types, consistent with its role as a functional immunosuppressive agent rather than a mere metabolic waste product (32). Colegio et al. demonstrated that conditioned medium from glycolytic tumour cells, and purified lactate at physiologically relevant concentrations, polarised macrophages toward an immunosuppressive, M2-like phenotype characterised by high arginase-1 and IL-10 expression (17). The mechanism involves lactate-mediated stabilisation of HIF-1*alpha* in macrophages via inhibition of prolyl hydroxylase domain enzymes, driving a transcriptional programme that overlaps with hypoxic M2 polarisation. More recently, the lactate receptor GPR81 (HCAR1) has been identified on macrophages, providing a receptor-level mechanism by which extracellular lactate can signal directly to the myeloid compartment independently of intracellular acidification (17).

Lactate suppresses T cell function through two mechanistically distinct pathways that compound one another. First, co-secretion of lactic acid and protons by MCT4 acidifies the extracellular TME (pH 6.5 to 6.9 in necrotic and pseudopalisading regions), impairing T cell IFN-*gamma* production, perforin secretion, and proliferative capacity in a pH-dependent manner (33, 34). Fischer et al. demonstrated that lactate at concentrations of 12.5 to 25 mM directly suppressed T cell proliferation and cytokine production through inhibition of NFAT nuclear translocation, while Dietl et al. showed that lactic acid, but not equimolar sodium lactate, inhibited TNF secretion and glycolysis in human monocytes, establishing the importance of the accompanying acidification (33, 34). Second, MCT1 (SLC16A1), expressed on effector T cells for lactate import, competes with MCT4-mediated tumour cell lactate export (35). High extracellular lactate loads the MCT1 import gradient in T cells, impairing their own lactate disposal and consequently their glycolytic flux, creating a direct metabolic competition for transporter capacity (22).

Lactate therefore occupies a unique and pivotal position within the glucose-glutamine-lactate competitive triad that defines the GBM suppressive ecosystem. Unlike glucose and glutamine, which are substrates whose depletion passively disadvantages competing cells, lactate is an active competitive signal produced in direct proportion to GBM glycolytic rate: the higher the glucose consumption by GBM cells, the more lactate is exported, and the more potently the myeloid compartment is polarised toward immunosuppressive M2 phenotypes. The third metabolite of the triad is not a passive consequence of the first but an amplifying paracrine effector. High glucose availability fuels GBM aerobic glycolysis, which simultaneously depletes glucose from T cells and generates the lactate signal that instructs myeloid cells to further suppress T cell function via GPR81/HIF-1*alpha* signalling. Disrupting glucose consumption reduces lactate production, withdrawing the M2-polarising paracrine signal, destabilising the myeloid immunosuppressive transcriptional programme, and reducing the downstream arginase-1-mediated amino acid depletion generated by M2-polarised macrophages. Interventions that reduce lactate production upstream, whether by PDK inhibition with dichloroacetate or by glucose restriction, therefore have immune consequences extending beyond direct tumour cell metabolic effects.

### 3.4 Amino acid depletion: arginase-1 and IDO1 as competitive immune suppressors

Beyond glucose and glutamine, tumour-associated myeloid cells and GBM cells deplete specific amino acids that are essential for T cell and NK cell function. Arginase-1, highly expressed in immunosuppressive MDSCs and M2-polarised macrophages within the GBM TME, catalyses the hydrolysis of L-arginine to ornithine and urea, depleting this substrate from the TME (24). T cells require arginine for TCR zeta-chain expression, proliferation, and effector cytokine production; arginase-1-mediated arginine depletion by myeloid cells therefore constitutes a direct competitive suppression of T cell function (24, 36, 37). Indoleamine 2,3-dioxygenase 1 (IDO1), expressed by GBM cells themselves and by tumour-associated myeloid cells, catalyses tryptophan catabolism to kynurenine, depleting tryptophan from the TME and generating kynurenine metabolites that activate the aryl hydrocarbon receptor (AhR) in T cells, driving Treg differentiation and suppression of effector T cell responses (38–40). GBM-specific evidence for IDO1 as a therapeutic target was provided by Wainwright et al., who demonstrated that combinatorial blockade of IDO, CTLA-4, and PD-L1 produced durable survival benefit in mice with GL261 brain tumours, with IDO inhibition required for the combination to reach full efficacy, establishing IDO1 as a non-redundant immunosuppressive mechanism in the GBM TME (41). The combined effect of arginase-1 and IDO1 in the GBM TME is to create an amino acid-depleted environment specifically hostile to T cell activation, expansion, and effector function while leaving the tumour and myeloid cells, which produce rather than consume these depleting enzymes, relatively unaffected.

### 3.5 Metabolic zonation within the GBM TME

The GBM TME is not metabolically uniform. Distinct anatomical zones, including the necrotic core, the pseudopalisading region surrounding necrosis, the enhancing tumour bulk, and the infiltrative margin, are characterised by dramatically different metabolic environments. The necrotic core and pseudopalisading zones are profoundly hypoxic, with HIF-1*alpha*-driven transcription maximally activated, glucose concentrations depleted, and lactate levels highest (5). The infiltrative margin, by contrast, is relatively well-perfused and retains closer proximity to normal brain metabolic substrate concentrations. Immune cell populations are distributed non-uniformly across these zones, with most T cell infiltration occurring at the tumour margin where metabolic conditions are less hostile, while immunosuppressive myeloid cells are enriched throughout the tumour core (26, 27). Spatial transcriptomic analyses using technologies such as Visium and MERFISH are beginning to map these metabolic-immune co-distributions at cellular resolution, revealing that the competitive suppressive programme is highest in the regions of greatest glycolytic activity and revealing potential therapeutic access points at the tumour-normal brain interface (27).

### 3.6 The competitive hierarchy: substrate abundance maintains suppression, substrate deprivation inverts it

In the glucose-and-glutamine-replete TME, GBM cells consume glucose and glutamine for rapid proliferation and biosynthesis; immunosuppressive myeloid cells consume the same substrates to maintain glycosylation-dependent immunosuppressive surface display, polyamine-dependent M2 transcriptional programmes, and ROS-generating effector functions; lactate produced by GBM glycolysis reinforces myeloid M2 polarisation through the GPR81/HIF-1*alpha* axis. T cells entering this environment are simultaneously substrate-starved, pH-suppressed, amino acid-depleted, and transporter-competed.

When the glucose-glutamine supply is disrupted, this coalition does not merely slow — it structurally disintegrates from multiple directions simultaneously. GBM cell biosynthetic capacity collapses as nucleotide synthesis, glutathione replenishment, and lipid production lose their carbon source. Myeloid immunosuppressive function deteriorates as hexosamine biosynthesis, polyamine production, and TCA-dependent suppressive metabolism are impaired. Lactate production falls, removing the GPR81/HIF-1*alpha*-driven M2 polarisation signal and destabilising the myeloid M2 transcriptional programme. T cells with retained FAO capacity entering this environment encounter reduced substrate competition, less extracellular acidification, reduced lactate-mediated transporter competition, and a myeloid compartment shifting toward more permissive phenotypes.

## 4 Fatty acid oxidation and the T cell metabolic competitive advantage

### 4.1 Effector versus memory T cell metabolism: the life cycle of metabolic flexibility

T cell activation drives a rapid shift from oxidative phosphorylation to aerobic glycolysis, mirroring the metabolic reprogramming of tumour cells. Activated effector CD8+ T cells upregulate GLUT1, hexokinase-2, and lactate dehydrogenase-A, adopting a glycolytic programme that fuels the rapid biosynthesis and cytokine secretion required for acute anti-tumour activity (42). However, as the acute effector response resolves and memory T cells are established, there is a fundamental metabolic shift: memory CD8+ T cells downregulate glycolytic enzymes, upregulate mitochondrial biogenesis, and shift their primary fuel source to fatty acid oxidation (24, 38). This metabolic flexibility is a critical distinction that requires precise qualification. The acute cytotoxic function of CD8+ effector T cells, including rapid perforin and granzyme secretion and clonal burst expansion, is glycolysis-dependent and cannot be fully substituted by FAO. FAO supports T cell metabolic resilience, mitochondrial spare respiratory capacity, long-term survival, and the sustained effector capacity of memory-phenotype T cells operating at reduced biosynthetic demand; it does not substitute for glycolysis in cells undergoing peak acute cytotoxic responses. The therapeutic relevance of FAO in this framework therefore lies in the persistence of T cells through a period of metabolic TME remodelling and their re-engagement with antigen once the competitive hierarchy has been inverted, rather than in sustaining maximal glycolytic killing capacity during the suppressive phase.

Pre-existing tumour-infiltrating T cells in an unmodified GBM TME represent a compromised population. Scharping et al. demonstrated that the TME directly suppresses T cell mitochondrial biogenesis, reducing spare respiratory capacity and FAO activity in tumour-infiltrating CD8+ T cells relative to peripheral T cells from the same host (43). Bengsch et al. showed that PD-1 signalling impairs both glycolytic and mitochondrial bioenergetic capacity simultaneously, establishing exhaustion as a metabolic state, not solely a transcriptional one (12). FAO-based metabolic fitness therefore applies primarily to T cells entering a metabolically reprogrammed TME: vaccine-primed effectors, adoptively transferred lymphocytes, or endogenously recruited T cells mobilised following myeloid repolarisation. The therapeutic sequencing rationale, in which metabolic TME remodelling precedes immune activation, is designed to present a metabolically permissive environment to T cells retaining intact mitochondrial capacity.

### 4.2 TRAF6/AMPK signalling and mitochondrial spare respiratory capacity

Pearce et al. demonstrated that TRAF6-deficient mice failed to form functional memory CD8+ T cells following acute infection (24). TRAF6-deficient T cells showed reduced mitochondrial fatty acid oxidation and could not be rescued by exogenous IL-15 alone, but were rescued by the Complex I inhibitor metformin, which inhibits mitochondrial Complex I, reducing OXPHOS efficiency and activating AMPK signalling that upregulates FAO as a compensatory energy source (24). The TRAF6-AMPK-PGC-1*alpha* signalling axis coordinates mitochondrial biogenesis and FAO enzyme expression during memory T cell differentiation. Mitochondrial spare respiratory capacity (SRC), defined as the difference between maximal and basal mitochondrial oxygen consumption, is the parameter most predictive of memory T cell functional survival (38, 44). Van der Windt et al. demonstrated that memory CD8+ T cells have substantially greater SRC than effector T cells, and that this SRC is maintained through FAO and CPT1A-dependent long-chain fatty acid import into the mitochondria (38). CPT1A expression therefore serves as a functional marker of metabolically fit T cells capable of sustaining activity in a glucose-poor, lipid-available environment such as the ketotic GBM TME created by pharmacological intervention.

### 4.3 Why FAO cannot sustain aggressive proliferation: the biosynthetic carbon problem

The fundamental reason that neither bulk GBM cells nor aggressively proliferating glioma stem cells can sustain rapid division through fatty acid oxidation is not energetic but biosynthetic. Rapid cell proliferation demands the continuous synthesis of nucleotides (requiring glucose-derived ribose-5-phosphate from the pentose phosphate pathway and glutamine as the nitrogen donor for purine and pyrimidine ring assembly), membrane lipids (requiring malonyl-CoA from acetyl-CoA and NADPH from the pentose phosphate pathway for *de novo* fatty acid synthesis), non-essential amino acids including serine and glycine (from 3-phosphoglycerate) and aspartate (from TCA oxaloacetate), and the bulk biomass required for daughter cell production. FAO yields acetyl-CoA and reducing equivalents (NADH, FADH2) for ATP synthesis but does not generate ribose-5-phosphate, does not provide the glutamine-nitrogen required for nucleotide ring assembly, and does not supply the reductive biosynthesis intermediates that glucose and glutamine jointly provide (13, 14). A rapidly dividing cell is therefore metabolically constrained to use glucose and glutamine as its primary biosynthetic carbon sources regardless of its FAO capacity. Raising FAO flux cannot substitute for this biosynthetic requirement; it can only supplement the ATP budget in a cell whose biosynthetic needs are already being met by glucose and glutamine.

The same biosynthetic constraint governs glioma stem cells when they exit quiescence. The transition from quiescent to aggressively proliferating GSC is accompanied by upregulation of glycolysis and glutaminolysis: glucose-derived ribose-5-phosphate and glutamine-nitrogen are required for nucleotide synthesis, and neither is supplied by FAO. G/Q restriction prevents this transition, confining GSCs to a slow-cycling state incapable of driving the growth and infiltration that characterise GBM recurrence. Studies using etomoxir to probe CPT1 dependence in GBM cells require methodological caution: Divakaruni et al. established that etomoxir above 40 micromolar causes CoA sequestration independent of CPT1A inhibition (45), and most published GBM etomoxir studies employ suprathreshold concentrations (46).

Memory T cell effector function, by contrast, is energetically demanding but biosynthetically modest: sustained cytokine secretion, directed cytotoxicity, and maintenance of spare respiratory capacity do not require the nucleotide and lipid biosynthetic throughput of a dividing cell. FAO-fuelled oxidative phosphorylation supports these programmes, and mitochondrial spare respiratory capacity provides the reserve to sustain function under metabolic stress (24, 38). Glucose and glutamine restriction therefore selectively impairs rapidly proliferating cells, which cannot divide without biosynthetic carbon from these substrates, while leaving intact the energetic requirements of T cell effector function that FAO can meet.

### 4.4 Regulatory T cells and FAO

*FOXP3*, the master transcription factor of regulatory T cells, was demonstrated by Angelin et al. to directly repress c-Myc and upregulate FAO-related gene expression, enabling Tregs to maintain their suppressive programme under low-glucose, high-lactate conditions (25). Michalek et al. and Gerriets et al. established that Treg suppressive function is preferentially supported by FAO and oxidative phosphorylation rather than glycolysis (47, 48). Treg expansion in the GBM TME is driven by tumour-derived TGF-*beta*, IL-10, and by lactate-dependent HIF-1*alpha* stabilisation that promotes *FOXP3* expression (17, 25). Disrupting tumour glycolysis removes this lactate-dependent polarising signal, reducing the TME input driving Treg expansion independently of FAO substrate availability. Angelin et al. demonstrated that Tregs in low-glucose environments show reduced proliferative capacity, such that glucose restriction limits Treg expansion rather than augmenting it (25).

### 4.5 M2 Macrophages, FAO, and the causal role of lactate

FAO is a feature of the M2 macrophage metabolic state, not the driver of M2 polarisation. M2 polarisation is instructed by IL-4, IL-13, and, in the GBM TME, by lactate acting via GPR81/HIF-1*alpha*; the FAO phenotype follows from STAT6-mediated upregulation of PPARgamma/delta and FAO enzyme expression downstream of cytokine receptor engagement (17). Tannahill et al. established that succinate is the pro-inflammatory TCA metabolite stabilising HIF-1*alpha* and driving IL-1*beta* in M1 macrophages (49). Itaconate, produced from cis-aconitate by IRG1/ACOD1 in M1 macrophages, functions as an anti-inflammatory brake on this programme and should not be conflated with succinate. Removing the lactate signal by disrupting tumour glycolysis withdraws the principal M2-polarising input in GBM regardless of lipid substrate availability. Leone et al. observed macrophage reprogramming toward inflammatory phenotypes following glutamine antagonism in association with reduced TME lactate, consistent with this mechanism (30).

### 4.6 Beta-hydroxybutyrate as T cell fuel, HDAC inhibitor, and myeloid reprogramming signal

The principal ketone body produced during pharmacological or endogenous ketosis, beta-hydroxybutyrate (*beta*-OHB), has multiple mechanisms of action relevant to the metabolic cell competition framework. As an oxidisable substrate, *beta*-OHB can be converted via SCOT (succinyl-CoA:3-oxoacid CoA transferase) to acetyl-CoA and used by mitochondria in place of glucose-derived pyruvate, supporting ATP production in T cells and neurons under conditions of glucose restriction. This provides an alternative metabolic fuel that may help T cells maintain energetic sufficiency in a glucose-depleted TME without relying on FAO of stored lipids.

Shimazu et al. demonstrated in a landmark 2013 Science publication that *beta*-OHB acts as an endogenous inhibitor of class I and class IIa histone deacetylases (HDAC1/2/3/8), binding the active site zinc ion and reducing HDAC activity at physiologically achievable circulating concentrations of 1 to 2 mM, as occur during therapeutic ketosis (50). HDAC inhibition by *beta*-OHB increases histone H3K9 and H3K14 acetylation at gene loci encoding antioxidant enzymes including FOXO3A and MnSOD, reducing oxidative stress in cells exposed to nutrient restriction. In T cells, HDAC inhibition has been associated with enhanced effector gene expression, improved memory formation, and augmented cytotoxic activity against tumour cells, adding an epigenetic dimension to the metabolic advantage conferred by ketosis. Additionally, Youm et al. demonstrated that *beta*-OHB blocks NLRP3 inflammasome activation in macrophages by preventing potassium efflux, reducing IL-1*beta* and IL-18 secretion (51). While NLRP3 activation in the GBM TME is complex and context-dependent, suppression of excessive inflammasome signalling in tumour-associated myeloid cells may contribute to the shift from a pro-tumorigenic inflammatory state toward a more homeostatic phenotype. Collectively, *beta*-OHB functions as a multi-target metabolic-epigenetic modulator that augments the immune benefits of glucose restriction beyond simple substrate competition.

### 4.7 Metabolic engineering of T cell competitive fitness

Engineering T cells intrinsically to favour oxidative metabolism over glycolysis increases their capacity to function in the nutrient-restricted, lactate-rich, and acidified GBM microenvironment. T cells with enhanced mitochondrial biogenesis, greater FAO capacity, and reduced glycolytic dependency are better positioned to sustain effector function under conditions that disadvantage glycolysis-dependent programmes. This applies to chimeric antigen receptor T cells and to ex vivo expanded tumour-specific lymphocytes.

The costimulatory domain incorporated into CAR T cell constructs exerts a profound and underappreciated influence on the metabolic programme of the engineered cell. Kawalekar et al. demonstrated that CAR T cells incorporating the 4-1BB (CD137) intracellular signalling domain displayed substantially enhanced mitochondrial biogenesis, greater oxidative phosphorylation and fatty acid oxidation activity, and adopted a central memory T cell phenotype compared with CAR T cells incorporating the CD28 costimulatory domain, which preferentially adopted an effector memory phenotype sustained by aerobic glycolysis (52). This metabolic divergence translated into superior *in vivo* persistence and improved antitumour activity, consistent with the prediction of the competitive framework: T cells biased toward OXPHOS and FAO outcompete glycolysis-dependent T cells in nutrient-restricted environments. The metabolic profiles conferred by 4-1BB and CD28 signalling domains therefore constitute a design choice with direct competitive fitness implications for CAR T cells intended to operate in the GBM TME, and 4-1BB is mechanistically preferable on metabolic grounds for this application.

Glycolytic restraint during ex vivo T cell expansion is a second mechanistically grounded engineering strategy. Sukumar et al. demonstrated that inhibiting glycolysis during CD8+ T cell priming, using either 2-deoxyglucose or low-dose rapamycin-mediated mTOR suppression, forced T cells to rely on oxidative metabolism during the priming phase, resulting in the generation of cells with a central memory phenotype, higher mitochondrial spare respiratory capacity, and superior long-lived antitumour activity following adoptive transfer (53). This finding converges with the established observation that high mTOR activity during effector T cell differentiation promotes terminal effector fate at the expense of memory formation (Ref 54, 55), and places glycolytic restraint during ex vivo expansion as a readily implementable modification to adoptive cell therapy manufacturing protocols. For GBM-directed cell therapies, restricting glycolysis during ex vivo expansion would produce T cells with intrinsically greater FAO capacity and mitochondrial fitness, metabolically matched to the glucose-restricted environment created by the therapeutic interventions described in Section 5.

Pharmacological augmentation of mitochondrial biogenesis through PGC-1*alpha* activation represents a third avenue. Chamoto et al. demonstrated that bezafibrate, a pan-PPAR agonist that activates PGC-1*alpha* and mitochondrial biogenesis, synergised with PD-1 blockade to produce durable antitumour responses in murine models at doses at which neither agent alone was efficacious (56). Mitochondrial activation via PGC-1*alpha* directly amplifies the FAO-based competitive advantage described in Section 4.2, increasing the SRC that enables T cells to sustain effector function under metabolic stress. Complementary approaches include CPT1A overexpression as a gain-of-function strategy to enhance mitochondrial long-chain fatty acid import capacity, and the expression of FOXO1, a transcription factor that promotes T cell quiescence, long-term survival, and memory gene expression while suppressing terminal effector differentiation, thereby extending the functional lifespan of adoptively transferred cells in the hostile GBM TME. Neither CPT1A overexpression nor exogenous FOXO1 expression has been evaluated in a clinically relevant GBM cell therapy model, and these remain experimental hypotheses requiring systematic preclinical validation. Collectively, the convergence of costimulatory domain selection, glycolytic restraint during manufacturing, and mitochondrial activation strategies identifies metabolic engineering as a mechanistically coherent approach to increasing the intrinsic competitive fitness of therapeutic T cells, complementary to, and conceptually inseparable from, the TME-directed metabolic reprogramming strategies described in Sections 3 and 5.

Prioritisation of these engineering strategies for GBM clinical translation requires distinguishing those with direct GBM preclinical validation from those with only non-GBM mechanistic support. Among currently described approaches, enforced GLUT1 overexpression has the most direct GBM-specific evidence: Shi et al. demonstrated that IL13R*alpha*2-BBz CAR-T cells overexpressing GLUT1 prolonged survival in an orthotopic GBM model and exhibited decreased exhaustion marker expression relative to unmodified controls, providing proof-of-concept that augmenting glucose uptake capacity in CAR-T cells directly counteracts competition from the glucose-avid GBM TME (57). This approach is distinct in mechanism from 4-1BB domain selection (which increases FAO and mitochondrial biogenesis) and from ex vivo glycolytic restraint (which shifts memory/effector balance), and the three strategies are not mutually exclusive; combining 4-1BB-based CAR design with GLUT1 co-expression addresses both competitive glucose acquisition and long-term oxidative fitness simultaneously. The most underexplored and potentially highest-yield opportunity is the combination of metabolic engineering with pharmacological TME remodelling: DON-mediated glutamine antagonism, by reprogramming TAMs away from the immunosuppressive phenotype and reducing myeloid glutamine competition, creates a more permissive environment into which GLUT1- or 4-1BB-optimised CAR-T cells can be delivered. SGLT2 inhibitor-mediated glucose restriction of GBM cells similarly reduces the competitive glucose sink against which engineered T cells must contend. The design of trials combining pharmacological metabolic reprogramming with metabolically engineered adoptive cell therapy represents the logical next step for GBM immunometabolic medicine and should be incorporated into window-of-opportunity clinical designs with paired metabolic imaging endpoints.

## 5 Therapeutic exploitation of metabolic cell competition

### 5.1 Pharmacological metabolic interventions

The competitive metabolic framework described in Sections 3 and 4 identifies several pharmacological strategies through which the glucose-glutamine-lactate suppressive axis can be disrupted. Glutamine antagonism with DON and its prodrugs JHU083 and JHU395 has the strongest mechanistic anchor: Leone et al. demonstrated *in vivo* that pan-glutamine antagonism reprogrammed TAMs from immunosuppressive to inflammatory phenotypes, restored CD8+ T cell infiltration, and produced survival benefit in a murine GBM model, with T cells — but not tumour cells — adapting metabolically by upregulating FAO (30). Clinical development of parent DON was historically constrained by dose-dependent gastrointestinal toxicity, predominantly nausea and emesis attributable to intestinal glutamine antagonism; JHU083 and JHU395 were designed to preferentially activate within the low-pH, esterase-rich TME to limit systemic glutamine antagonism (58), though gastrointestinal tolerability at immunologically active doses requires prospective evaluation in human GBM trials. Glucose restriction is achievable through SGLT2 inhibitors (with direct pharmacodynamic proof of target engagement in human GBM (59–61)), which additionally promote pharmacological ketosis and elevation of beta-hydroxybutyrate, an endogenous HDAC inhibitor that augments T cell effector gene accessibility (50, 51, 62, 63); however, the assumption that pharmacological ketosis is uniformly detrimental to GBM requires qualification: OXCT1 (encoding SCOT, the rate-limiting enzyme of ketone oxidation) is variably expressed across cancer cell lines, and preclinical evidence indicates that some brain tumour cells can utilise beta-hydroxybutyrate as a supplementary oxidative substrate, potentially limiting the selective metabolic disadvantage conferred by ketosis; patient or tumour stratification by OXCT1 expression or ketone oxidative capacity may therefore be necessary to identify tumours in which ketosis is net-suppressive. Lactate production can be reduced upstream by pyruvate dehydrogenase kinase inhibition with dichloroacetate, which reactivates the pyruvate dehydrogenase complex in glycolytic tumour cells and reduces lactate output at its biosynthetic source (64–66); lactate export can be targeted downstream by MCT inhibitors, though MCT1-selective inhibition carries the immunological concern of impairing T cell lactate disposal and MCT4-selective agents are preferable (67); GBM-specific immunological data for MCT inhibitors are presently absent and represent a critical gap in the translational evidence base. Pharmacological ascorbate exploits the GLUT1 overexpression of glycolytic GBM cells to deliver dehydroascorbate selectively, inactivating GAPDH through oxidative stress while additionally destabilising HIF-1*alpha* via prolyl hydroxylase reactivation (68–70). Sodium phenylbutyrate provides modest glutamine depletion through urinary phenylacetylglutamine excretion combined with sub-stoichiometric HDAC inhibitory activity, though the pharmacokinetics of butyrate exposure at clinically tolerated doses may be insufficient for robust HDAC inhibition *in vivo (*71, 72). Mebendazole demonstrates multi-target anti-tumour activity via tubulin polymerisation inhibition, VEGFR2/PDGFR inhibition, and Bcl-2 downregulation, with survival benefit in two immune-competent GBM preclinical models (73); whether direct glutamine antagonism contributes to this activity has not been established in peer-reviewed publications and cannot currently be incorporated into the metabolic competition framework. None of these strategies has been evaluated in a randomised GBM trial within an immune-competent metabolic priming framework, all face the fundamental confounder of corticosteroid-induced hyperglycaemia and lymphopenia, and all require biomarker-stratified window-of-opportunity clinical designs with paired metabolic imaging and immune profiling. Mechanisms, evidence levels, GBM-specific data, and principal caveats for each intervention are summarised in **Table 1**.

**Table 1:** Summary of pharmacological metabolic interventions targeting the GBM tumour microenvironment metabolic competition axis.

Agent / intervention	Primary mechanism	Target pathway	Evidence level	GBM-specific data and gaps
DON / JHU083 / JHU395	Pan-glutamine amidotransferase antagonism; prodrugs activated in TME	Glutamine competition; myeloid repolarisation; T cell metabolic rescue	Preclinical (strong); Phase I ongoing	Leone 2019 (Science): GL261 model, TME reprogramming. CNS penetration of prodrugs requires validation. No randomised GBM trial.
Sodium phenylbutyrate	Glutamine depletion via phenylacetylglutamine excretion; HDAC I/IIa inhibition	Glutamine competition; epigenetic T cell augmentation; tumour differentiation	Preclinical; Phase I/II (other cancers)	Dose-dependent glutamine depletion unvalidated at oncology doses in GBM. No randomised immunotherapy combination trial.
SGLT2 inhibitors	Renal glucose reabsorption blockade; indirect ketogenesis promotion; direct Complex I inhibition (canagliflozin)	Glucose competition; insulin/IGF-1 reduction; beta-OHB elevation	Mechanistic; Phase I pharmacodynamic (human GBM; n=5)	Wright 2018: SGLT2 protein confirmed in GBM cells and tumour neovasculature by immunocytochemistry; Me4FDG PET demonstrates SGLT-mediated uptake 14-fold above normal brain. Wright 2024: single-dose empagliflozin reduces GBM Me4FDG uptake in 5/5 patients; in vivo target engagement confirmed. Dexamethasone confounds glucose restriction.
Mebendazole	Tubulin polymerisation inhibition; VEGFR2/PDGFR inhibition; Bcl-2 downregulation	Anti-proliferative; anti-angiogenic; indirect TME remodelling	Preclinical (GBM); Phase II feasibility	Bai 2011 (Neuro Oncol): survival benefit in GL261 and PDGF-driven GBM models. Direct glutamine antagonism activity not established in peer-reviewed literature. Metabolic competition mechanism requires dedicated in vivo investigation.
MCT1/MCT4 inhibitors (AZD3965, syrosingopine)	Blockade of lactate export from glycolytic tumour cells; reduction of extracellular lactate	Lactate-mediated M2 polarisation reversal; T cell pH restoration	Preclinical; AZD3965 Phase I (non-CNS)	CNS penetration and GBM-specific immunological data lacking. Rational mechanistic target; clinical translation pending.
Dichloroacetate (DCA)	PDK1/2 inhibition; reactivates PDC; diverts pyruvate to TCA; reduces lactate production; increases mitochondrial ROS; inhibits HIF-1alpha	Upstream lactate reduction (complementary to MCT inhibitors); HIF-1alpha suppression; apoptosis via mitochondrial depolarisation; engages GSC compartment	Preclinical; Phase I/II (GBM, n=5)	Michelakis 2010 (Sci Transl Med): Warburg reversal in biopsies; apoptosis in CD133+/nestin+ GSCs; PDK2 overexpressed in all GBM tested. Dose-limiting toxicity: peripheral neuropathy.
IV Pharmacological Ascorbate	DHA/GLUT1 uptake depletes GSH; ROS inactivate GAPDH; PHD reactivation destabilises HIF-1alpha; extracellular H2O2 pro-oxidant; TET cofactor for T cell epigenetic reprogramming	GLUT1-selective glycolytic vulnerability; HIF-1alpha suppression; direct cytotoxicity; T cell effector gene demethylation	Preclinical; Phase I (GBM)	Allen 2019 (Clin Cancer Res): Phase I, n=11, no DLT, median OS 18 months. G6PD deficiency: absolute contraindication. Dexamethasone-GLUT competition pharmacology unstudied.
Valganciclovir	HCMV UL54 (DNA polymerase) inhibition; suppresses IE1/IE2-mediated c-Myc transactivation; reduces cmvIL-10-driven M2 polarisation; reduces US28-mediated chemokine scavenging	HCMV-amplified glycolysis (c-Myc/GLUT1/LDHA/PKM2) and glutaminolysis (c-Myc/GLS1/ASCT2); increased lactate-driven M2 polarisation; myeloid M2 reinforcement via cmvIL-10; T cell chemotactic exclusion via CCL5/CX3CL1 scavenging	Preclinical mechanistic (HCMV infection models); Phase II retrospective and add-on trial	Stragliotto 2013: retrospective OS benefit in HCMV-positive GBM. Söderström 2020 (Clin Cancer Res): add-on trial survival signal. Randomised double-blind trial (n=220, primary endpoint OS) ongoing. HCMV prevalence in IDH-wildtype GBM varies by detection methodology; stratification by viral antigen burden required.

Evidence levels reflect current status as of 2026. AED, anti-epileptic drug; beta-OHB, beta-hydroxybutyrate; CNS, central nervous system; DON, 6-diazo-5-oxo-L-norleucine; GBM, glioblastoma; HDAC, histone deacetylase; IGF-1, insulin-like growth factor 1; MCT, monocarboxylate transporter; NLRP3, NOD-like receptor family pyrin domain-containing 3; SGLT2, sodium-glucose cotransporter 2; TME, tumour microenvironment; VEGFR2, vascular endothelial growth factor receptor 2.

## 6 An integrated model of metabolic cell competition in GBM: controversies and research gaps

### 6.1 The metabolic fitness hierarchy: a synthesised model

The GBM TME is organised around a metabolic fitness hierarchy sustained by two interlocking mechanisms. GBM cells and immunosuppressive myeloid cells consume glucose and glutamine at high rates, depleting these substrates from the shared niche. Lactate and amino acid depletion products generated by this consumption act as paracrine suppressive signals that further impair infiltrating T cells through GPR81/HIF-1*alpha*-mediated myeloid repolarisation, extracellular acidification, and monocarboxylate transporter competition. Disrupting glucose and glutamine availability dismantles both mechanisms simultaneously: GBM biosynthetic capacity falls, myeloid immunosuppressive programmes lose their metabolic substrate, and lactate-driven M2 polarisation is withdrawn. T cells retaining FAO capacity enter a metabolically less hostile environment as a consequence.

### 6.2 Glioma stem cells and substrate restriction

Aggressive GSC behaviour is coupled to upregulated glycolysis and glutaminolysis: the biosynthetic demands of rapid proliferation, including nucleotide synthesis and lipid production, cannot be met through alternative substrates. G/Q restriction therefore prevents the phenotypic transition that produces clinical harm. Quiescent GSCs in this setting are slow-cycling and do not contribute meaningfully to tumour progression. The operative clinical variable is whether G/Q restriction can be sustained long enough for immune-mediated clearance to reduce the viable tumour burden before GSCs re-enter the proliferative state. Combining metabolic restriction with effective immune activation addresses both the bulk tumour compartment and the stem cell reservoir.

### 6.3 Spatial heterogeneity and the tumour-brain interface

The metabolic competition framework operates differently across GBM anatomical zones. The necrotic core and pseudopalisading zone, characterised by extreme hypoxia and maximal glycolytic activity, represent the environment in which metabolic competition is most intense and in which T cell exclusion is most complete (5). The infiltrative margin, where tumour cells invade relatively well-perfused normal brain parenchyma, is metabolically distinct and represents both the site of greatest T cell access and the primary source of tumour recurrence. Spatial metabolomic and transcriptomic approaches, including the multi-omic spatial atlas of GBM published by Ravi et al., have begun to reveal that the tumour-host interface is characterised by distinctive bidirectional metabolic exchanges not captured by bulk tumour analysis (27). Therapeutic strategies must account for this spatial heterogeneity: a systemic metabolic intervention may adequately modulate the metabolic environment at the tumour margin while having limited access to the deeply hypoxic core. Drug delivery strategies combining metabolic agents with methods to improve tumour penetration, such as focused ultrasound-mediated blood-brain barrier opening, may be required to achieve adequate intratumoral drug concentrations.

### 6.4 Cytomegalovirus as metabolic amplifier of the competitive coalition

Human cytomegalovirus (HCMV) is detected in a substantial proportion of IDH-wildtype GBMs and provides a second, virally driven mechanism through which the metabolic competitive hierarchy is amplified (74). The viral immediate-early proteins IE1 and IE2 are promiscuous transcriptional transactivators that directly transactivate the c-*Myc* promoter via basal promoter elements within hours of infection (75). Elevated c-*Myc* drives both arms of the glucose-glutamine competitive axis simultaneously. On the glycolytic arm, c-*Myc* directly upregulates GLUT1, LDHA, and PKM2, increasing glucose uptake, glycolytic flux, and lactate production — amplifying the paracrine GPR81/HIF-1*alpha* M2 polarisation signal. On the glutamine arm, c-*Myc* suppresses miR-23a and miR-23b, releasing post-transcriptional inhibition of mitochondrial glutaminase (GLS1), and directly upregulates ASCT2 (SLC1A5) via E-box elements in its promoter, increasing glutamine import capacity (76). HCMV infection therefore amplifies the entire metabolic competitive axis through a single viral transcriptional effector, simultaneously increasing glucose consumption, lactate output, and glutamine demand in the host tumour cell (77).

At the myeloid level, HCMV independently reinforces immunosuppression through mechanisms that operate in parallel with the lactate-GPR81/HIF-1*alpha* axis. The viral cytokine cmvIL-10 (encoded by UL111A) directly drives M2 macrophage polarisation in infected monocyte-lineage cells. The virally encoded chemokine receptor US28, constitutively expressed in infected cells and secreted on exosomes into the TME, scavenges CCL5 and CX3CL1 at high affinity, preventing T cell recruitment and retention independent of metabolic substrate availability. The competitive coalition is therefore reinforced at two levels simultaneously by CMV: tumour cell metabolic competitive capacity is increased, and the myeloid immunosuppressive compartment is polarised through a mechanism orthogonal to lactate signalling.

Valganciclovir, a prodrug of ganciclovir that inhibits HCMV DNA polymerase (UL54), addresses all three arms by suppressing viral replication: IE1/IE2 expression is reduced, c-*Myc* transactivation falls, GLS1 and ASCT2 upregulation is withdrawn, cmvIL-10 secretion declines, and US28 exosomal release is reduced. Retrospective and add-on trial data support a survival signal with valganciclovir in HCMV-positive GBM (78). A randomised double-blind trial (n=220, primary endpoint overall survival) initiated in 2019 has not yet reported final results. The prevalence of HCMV in IDH-wildtype GBM varies across studies depending on detection methodology and contamination controls; CMV seroprevalence and intratumoral viral antigen burden should be incorporated as stratification variables in metabolic immunotherapy trial design.

### 6.5 Corticosteroids and patient stratification

This review is scoped to *IDH*-wildtype GBM, which under the 2021 WHO classification constitutes the defining entity of the disease. *IDH*-mutant grade 4 astrocytoma is a biologically and metabolically distinct entity whose oncometabolite-driven immune suppression mechanisms — including 2-HG-mediated inhibition of alpha-ketoglutarate-dependent dioxygenases and consequent T cell trafficking impairment — are not the subject of this review and require separate evaluation. Within *IDH*-wildtype GBM, patient stratification in metabolic immunotherapy trials must incorporate *MGMT* promoter methylation status, corticosteroid dose and duration, and baseline immune profiling. Dexamethasone confounds glucose metabolism, lymphocyte trafficking, and T cell compartment size in ways that directly undermine the metabolic competition interventions reviewed here; prospective steroid-minimisation protocols are therefore a necessary element of trial design.

### 6.6 The T cell source requirement

Realising the metabolic competitive advantage of T cells in a reprogrammed TME requires a source of metabolically competent T cells. Three candidate sources are relevant: endogenously expanded tumour-reactive T cells primed by therapeutic vaccination; adoptively transferred tumour-specific lymphocytes expanded ex vivo; and peripheral T cells recruited following myeloid repolarisation. Each faces distinct constraints. Therapeutic vaccination in GBM has produced inconsistent results related to antigen heterogeneity and TME immunosuppression. Adoptive cellular therapy has encountered poor engraftment and limited trafficking across the blood-brain barrier. Endogenous T cell recruitment depends on successful myeloid repolarisation clearing the physical and metabolic barriers at the tumour margin.

Existing preclinical metabolic competition studies have largely evaluated metabolic interventions in immunologically intact syngeneic hosts rather than in models that characterise the T cell source requirements separately. Key unanswered questions include whether metabolic TME remodelling through glutamine antagonism, glucose restriction, or MCT inhibition is sufficient to restore T cell trafficking into previously excluded tumour regions, and whether T cells entering a metabolically reprogrammed GBM TME maintain sufficient functional competence for anti-tumour cytotoxicity. These questions are directly relevant to the metabolic engineering strategies described in Section 4.7, which are designed to maximise the intrinsic fitness of the T cell source used.

### 6.7 Technology gaps and clinical trial design priorities

Several technologies are required to evaluate metabolic competition interventions in human GBM. *In vivo* metabolic flux measurement using hyperpolarised ^13^C MR spectroscopy of labelled pyruvate or glutamine would enable non-invasive assessment of glycolytic activity and glutamine flux in the TME, and their response to intervention. Spatial metabolomics applied to GBM surgical specimens, using DESI-MS, MALDI-MS, or inferred metabolic state from spatial transcriptomics, would characterise zonal metabolic heterogeneity at cellular resolution. Multi-parametric peripheral immune monitoring, including mass cytometry of circulating T cell and myeloid compartments and plasma metabolite profiling, provides pharmacodynamic biomarkers accessible without tumour re-biopsy. Clinical trials should incorporate biomarker-stratified window-of-opportunity designs with mandatory pre- and post-treatment tissue collection and paired metabolic imaging, permitting mechanism-based patient selection rather than empirical dose escalation.

## 7 Conclusion

GBM has resisted decades of therapeutic innovation in large part because treatment strategies have addressed individual molecular targets without engaging the ecosystem-level metabolic competition that sustains immune evasion. The central argument of this review is that GBM cells and immunosuppressive myeloid cells form a substrate-dependent competitive coalition: both require glucose and glutamine to maintain their respective proliferative and immunosuppressive programmes, and the lactate produced by GBM glycolysis directly reinforces myeloid M2 polarisation through GPR81/HIF-1*alpha* signalling, creating a paracrine loop in which tumour metabolism actively maintains the immunosuppressive microenvironment. T cells are excluded from this environment not merely by single-receptor suppression but by the combined weight of substrate starvation, pH toxicity, and lactate-driven myeloid programming. When the glucose-glutamine supply is disrupted, the coalition disintegrates at multiple nodes simultaneously, and the metabolic conditions for T cell function improve without any immune-directed intervention. The differential ability of memory T cells to sustain effector function through fatty acid oxidation, which does not require the biosynthetic carbon supply that rapid proliferation demands, defines the therapeutic asymmetry on which the entire framework rests.

Practical translational challenges remain substantial. Dexamethasone confounds glucose metabolism and T cell function in a manner directly antagonistic to metabolic immunotherapy, and prospective steroid-minimisation is a necessary trial design element. The requirement for metabolically competent T cells to exploit a reprogrammed TME is not addressed in existing preclinical literature, and the engineering strategies described in Section 4.7 represent candidate solutions requiring validation in GBM-relevant models. G/Q restriction confines GSCs to a slow-cycling, clinically indolent state by blocking the biosynthetic substrate required for aggressive proliferation; durability of that restriction is the clinical variable to be optimised. None of the pharmacological strategies in **Table 1** has been tested in a randomised, immune-competent, metabolically monitored GBM trial. Biomarker-stratified window-of-opportunity designs with metabolic imaging and paired pre- and post-treatment immune profiling are the study designs required to evaluate these interventions.
